# Editorial

**DOI:** 10.1002/osp4.158

**Published:** 2018-02-20

**Authors:** D. B. Sarwer

**Affiliations:** ^1^ Obesity Science & Practice

I write my editorial for Volume 4, Issue 1 of *Obesity Science & Practice* with great enthusiasm, excited to update you on the journal's accomplishments and plans for the future. For the past 2 years, we have published four issues in both Volumes 2 and 3 of the journal. The issues have been composed of 10 to 12 papers on average, which have come from research teams from around the globe. Submissions to the journal continue to increase steadily, both in terms of papers sent directly to the journal and those referred from the other Wiley obesity journals – *Obesity*, *Obesity Reviews*, *Pediatric Obesity*, *Clinical Obesity and Diabetes*, and *Obesity & Metabolism*. As a strong statement of our early success, Volume 4 is scheduled to include six issues published throughout 2018. The journal's leadership team is very pleased with the move to six issues and believes it represents another strong statement about our early success and potential.

I would like to highlight some other accomplishments. Our readership increased 83% from 2016 to 2017. Five of our papers were downloaded over a thousand times in 2017. The two most downloaded papers ‘Liraglutide for Weight Management: A Critical Review of the Evidence’ by Mehta and colleagues and ‘Equivalent Reductions in Body Weight During the Beef WISE Study: Beef's Role in Weight Improvement, Satisfaction, and Energy’ by Sayer and colleagues were published in 2017 and downloaded more than 2,000 times. Three papers, ‘Associations Between Feeding Practices and Maternal and Child Weight Among Mothers Who Do and Do Not Correctly Identify Their Child's Weight Status’, ‘Alternate‐day Versus Daily Energy Restriction Diets, Which is More Effective for Weight Loss? A Systematic Review and Meta‐analysis’ and ‘Adherence to Low‐Carbohydrate and Low‐Fat Diets in Relation to Weight Loss and Cardiovascular Risk Factors’, were our top papers with regard to Altmetric scores, reflecting the highest level of attention from the mainstream media, public policy documents, social and academic networks and post‐publication peer‐review forums.

Looking forward, *Obesity Science & Practice's* homepage will have a new look later in 2018. We also plan to review and expand our Editorial Board, with a specific focus on increasing representation from accomplished professionals from around the world. The current members have made significant contributions to the success of the past 3 years, and I hope that the inclusion of new members will lead to further success. Finally, we are eagerly expecting our index evaluation with Thomson Reuters later this year. I will share that with you as soon as we receive it.

As always, I remain grateful for the support from the editorial teams from the other Wiley obesity journals. The leadership teams from The Obesity Society and World Obesity remain instrumental to our success. Ms Lena Jacobsen, Journals Publishing Manager, and the rest of the Wiley publication team have been invaluable contributors to the day‐to‐day operations of the journal that have created our strong foundation. Finally, a thank you to our authors and readers. Without your manuscript submissions and readership, we would not have enjoyed the early success we have experienced. I hope you continue to turn to *Obesity Science and Practice* for high quality scholarship in our field throughout 2018 and in the years ahead.

